# The Mental Health Consequences of Traumatic Brain Injury Among Adults 50 and Older

**DOI:** 10.1093/geroni/igaf122.2274

**Published:** 2025-12-31

**Authors:** Laura Mehegan, G Rainville, Rachel Lazarus

**Affiliations:** AARP, Washington, District of Columbia, United States; AARP, Washington, District of Columbia, United States; AARP, Washington, District of Columbia, United States

## Abstract

In September 2024, AARP fielded a nationally representative survey to explore the prevalence and mental health outcomes among adults 50 and older who have experienced a traumatic brain injury (TBI) (N = 3,657). The identification of possible/probable TBI from nine different causes of head injury (e.g., falls, car wrecks, sports injuries) is based on a new scoring system developed by scientists at the Centers for Disease Control and Prevention (Daugherty et al., 2024). Previously, the prevalence of TBI was based on hospital records but the new scoring system identifies TBIs treated in other settings or not treated at all and are based on self-report. Overall, the results show that three in 10 (30%) adults 50-plus have likely experienced a TBI in their lifetime. Adults 50-plus who have had one or more possible/probable TBI at any point in their life fare worse on mental health outcomes -- higher levels of anxiety and depression and lower mental well-being scores -- compared to adults who have not experienced a head injury or have experienced a head injury unlikely to be TBI. Adults who have experienced multiple TBIs or a TBI after age 50 fare even worse in these mental health domains. Because adults in this age group are under-studied, this research represents a step forward in understanding the prevalence of TBI in this age group as well as the consequences for mental health outcomes. It also highlights the need for strategies to prevent TBI at any age with emphasis on fall prevention among older adults.

